# Design of an alternate antibody fragment format that can be produced in the cytoplasm of *Escherichia coli*

**DOI:** 10.1038/s41598-023-41525-3

**Published:** 2023-08-30

**Authors:** Aatir A. Tungekar, Lloyd W. Ruddock

**Affiliations:** Protein and Structural Biology Research Unit, Faculty of Biochemistry and Molecular Medicine, University of Oulu, 90220 Oulu, Finland

**Keywords:** Proteins, Immunochemistry, Expression systems, Molecular engineering

## Abstract

With increased accessibility and tissue penetration, smaller antibody formats such as antibody fragments (Fab) and single chain variable fragments (scFv) show potential as effective and low-cost choices to full-length antibodies. These formats derived from the modular architecture of antibodies could prove to be game changers for certain therapeutic and diagnostic applications. Microbial hosts have shown tremendous promise as production hosts for antibody fragment formats. However, low target protein yields coupled with the complexity of protein folding result in production limitations. Here, we report an alternative antibody fragment format ‘Fab_H_3’ designed to overcome some key bottlenecks associated with the folding and production of Fabs. The Fab_H_3 molecule is based on the Fab format with the constant domains replaced by engineered immunoglobulin G1 (IgG_1_) C_H_3 domains capable of heterodimerization based on the electrostatic steering approach. We show that this alternative antibody fragment format can be efficiently produced in the cytoplasm of *E. coli* using the catalyzed disulfide-bond formation system (CyDisCo) in a natively folded state with higher soluble yields than its Fab counterpart and a comparable binding affinity against the target antigen.

## Introduction

Among biotherapeutics, the monoclonal antibodies (Mabs) segment holds a dominant market position, however this trend appears to be gradually changing with the development of novel antibody formats and new medicines^1^. Full-length monoclonal antibodies have a long circulating half-life which might render them unsuitable for certain therapeutic and diagnostic applications^2^. As a potential solution, antibody fragments (Fabs) have emerged as key players in the biopharmaceutical industry. They offer certain advantages such as improved and deep tumor penetration, binding to specific epitopes inaccessible to Mabs, monovalent antigen binding with potentially reduced immunogenicity, higher stability than smaller antibody fragment formats and faster clearance^3–5^. A multitude of applications of antibody-based molecules as therapeutic and diagnostic agents have intensified their demand and subsequently the need for their production in high yields.

Many approved biosimilar and ‘me-too’ type products have been produced in microbial expression systems of which *E. coli* has remained a host of choice^6^. As Fab and scFv antibody fragments are non-glycosylated and relatively small, their production in *E. coli* can be simpler and more economically viable than the conventional mammalian cell culture systems. Antibody fragments are disulfide-bonded proteins which are traditionally recombinantly produced in the oxidizing periplasm or as inclusion bodies in the reducing cytoplasm of *E. coli*. However, both approaches pose critical bottlenecks in the efficient production of these proteins of interest. Periplasmic expression of Fabs often results in low protein yields, mainly due to inefficient protein translocation and the small volume of the periplasm, while cytoplasmic expression faces limitations in terms of protein degradation of the non-native state and aggregation to inclusion bodies. Of the seven marketed Fabs, two are produced in *E. coli* in the form of inclusion bodies^3,7^. Fab folding is a complex process, involving disulfide-bond formation, cis–trans prolyl isomerization and controlled oligomerization, and hence refolding of inclusions bodies is often inefficient and costly^8^.

**Figure 1:**
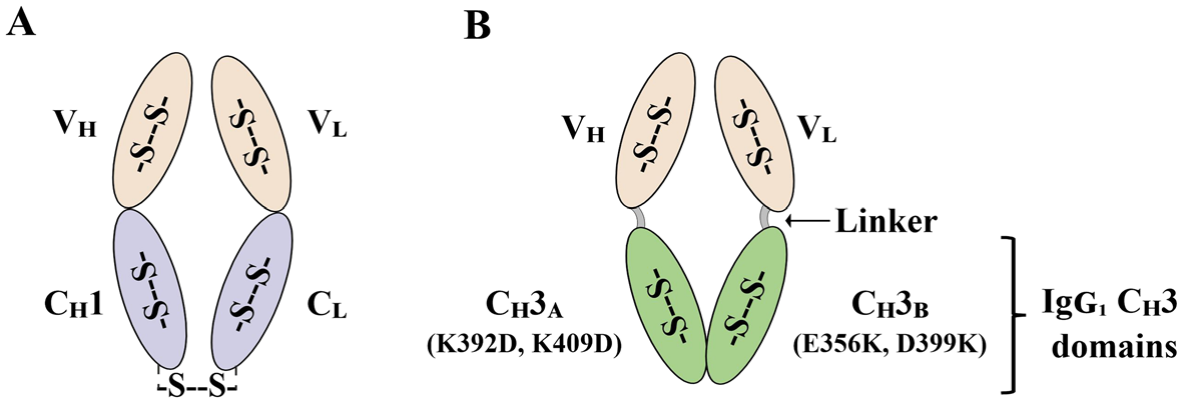
Schematic illustration of (**A**) Antibody fragment (Fab) and (**B**) Antibody fragment format with C_H_3 domains (Fab_H_3). Reversing symmetric charge complementarity at the C_H_3 domain interface allows the suppression of homodimerization due to unfavourable repulsive charge interactions thereby allowing the formation of a Fab like heterodimer.

Fab fragments are composed of two covalently linked polypeptide chains, namely the heavy (HC) and light (LC) chains which respectively contain two constant domains C_H_1 and C_L_ and two antigen-binding variable domains V_H_ and V_L_ (Fig. 1A). The C_H_1 domain is an intrinsically disordered protein in isolation and remains in an unfolded state irrespective of the formation of disulfide bonds^9^. One of the critical rate-limiting steps in the folding of antibody fragments is the association of the constant domains as the C_H_1 domain adopts the typical immunoglobulin (Ig) fold only on interaction with the C_L_ domain^9,10^. In addition, low HC stability coupled with formation of covalent and non-covalently linked LC dimers warrants the need for balancing the synthesis of the two chains^11,12^. Designing and screening constructs with varying translational strengths of the LC and HC for optimal Fab expression can be laborious, expensive and may not necessarily result in success. There have been several approaches to drive efficient heterodimerization in a Fab molecule, for example, Ojima-Kato et al. reported the fusion of leucine-zipper peptide pairs to the constant domains (Zipbody)^13^. However, this limits the therapeutic application of Fabs due to the requirement for tag cleavage steps and subsequent immunogenicity concerns.

Limitations associated with efficient Fab production raises the need for alternative antibody fragments which retain the beneficial properties of Fabs while overcoming their production bottlenecks. Here, we communicate an alternate antibody fragment format called ‘Fab_H_3’ in which the constant domains of a Fab molecule, namely REGN10987 (PDB ID: 6XDG) against the receptor binding domain (RBD) of SARS-CoV-2, are replaced by IgG_1_ heavy chain constant 3 (C_H_3) domains (Fig. 1B). This removes the limitations associated with the C_H_1 domain in Fabs. The rationale behind using IgG_1_ C_H_3 domains is their high solubility and stability coupled with their remarkable dimerization propensity^14,15^. As heterodimerization is a critical attribute of Fabs, we modified the IgG_1_ C_H_3 domains using the previously established ‘Charge-to-charge swap design’ which allows efficient non-covalent heterodimerization of the C_H_3 domains and allows the suppression of homodimerization though with a reduction in thermostability^16^. We report that the Fab_H_3 format can be produced solubly with comparatively higher yields than the wild type Fab molecule in the cytoplasm of *E. coli* in different strains and culture media. In addition, we show that the REGN10987-based Fab_H_3 format produced using the CyDisCo system is a natively folded heterodimer with a comparable affinity to its Fab counterpart against the SARS-CoV-2 RBD.

## Results

### Fab_H_3 design

A number of crystallizable fragment (Fc)-engineering approaches for the development of bispecific protein-based therapies based on C_H_3 heterodimerization have been reported in the past few decades^17–20^. For example, Wozniak-Knopp G. et al. reported the replacement of C_H_1 and C_L_ domains with covalently linked C_H_3 domains using the ‘knobs-into-holes’ (KiH) technology for a full-length trastuzumab molecule produced in HEK293-6E cells^21^. However, their resultant domain-exchanged monoclonal antibody showed low solubility and a four-fold weaker binding affinity to its target as compared to the parent trastuzumab. As well as the KiH technology to enable C_H_3 heterodimerization, the other widely used method is the asymmetric charge polarity approach. We chose to adopt this to test the Fab_H_3 format as it is reported to suppress homodimerization more efficiently than the ‘knobs-into-holes’ without the introduction of an additional disulfide bond^16,22^ as this may increase the oxidative folding related stress on the production system as well as increase the misfolding and aggregation propensity^23^.

It has been shown that the IgG_1_ C_H_3 domain interface consists of charged residues that interact through favorable electrostatic interactions surrounding a central hydrophobic core^16,24^. As the hydrophobic core plays an important role in protein folding and stability^25^, the electrostatic steering approach involves reversing the charge polarity on the rim of the core at the interface of the C_H_3 domains to favor heterodimerization over homodimerization^16^. In the Fab_H_3 construct we have designed, mutations K392D, K409D were introduced into the C_H_3 domain fused to the V_H_ domain and E356K, D399K were introduced into the C_H_3 domain fused to the V_L_ domain (nomenclature based on^16^; PDB ID: IL6X). To ensure sufficient flexibility and folding of the variable domains and to minimize steric hindrance during C_H_3 domain dimerization, the V_H_ and V_L_ domains of the target Fab were fused to the C_H_3 domains via flexible linkers composed of glycine and serine residues of varying length.

### Soluble expression of wild-type REGN10987 Fab and Fab_H_3

As the interfaces between the V_L_/V_H_ and C_H_3 domains are non-native, there is the potential for steric or other repulsions between them. While in silico modelling based on published structures suggested a linker as short as four amino acids could work, we wanted to experimentally validate the optimal inter-domain linker length. To do this, we compared the soluble expression of wild type REGN10987 Fab against Fab_H_3 constructs with varying linker lengths between the variable domains and C_H_3 domains from 4 to 17 amino acids in length (amino acid sequences in Supplementary Table S1).

Since all the proteins of interest are disulfide-bonded proteins, their soluble expression was tested in the presence of CyDisCo components in the cytoplasm of *E. coli*. CyDisCo is a catalyzed cytoplasmic disulfide formation system allowing the production of disulfide-bonded recombinant proteins in *E. coli*^26,27^. The sulfhydryl oxidase Erv1p (*S. cerevisiae*) catalyzes the oxidation of thiols on the protein to form disulfides and the isomerase PDI (*H. sapiens*) brings about the isomerization of non-native disulfides thus allowing the protein to be recombinantly produced in a natively folded disulfide-bonded state in the *E. coli* cytoplasm^28^.

**Figure 2:**
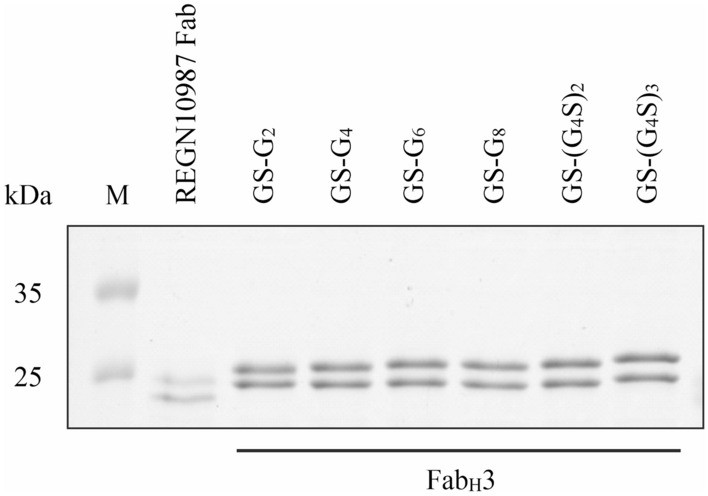
SDS-PAGE analysis of IMAC-purified REGN10987 Fab and Fab_H_3 produced solubly using CyDisCo in the cytoplasm of *E. coli*. Fab_H_3 variants with different linker lengths (G: Glycine, S: Serine) were compared to the wild type Fab to determine the influence on soluble protein yields (M: Protein Marker). See Supplementary Fig. S2 for the uncropped gel image.

SDS-PAGE analysis of immobilized metal affinity chromatography (IMAC) based purified proteins showed that the Fab_H_3 is efficiently produced in the form of a heterodimer with the two chains being present in a 1:1 ratio (Fig. 2). The Fab_H_3 format was produced in comparatively higher soluble yields than the REGN10987 wild-type Fab without any further process optimization (Fig. 2). The linker length did not influence the soluble yields of the Fab_H_3 format or the ratio between the chains. The linker length also did not influence the thermal stability of Fab_H_3 (as determined by Nano Differential Scanning Fluorimetry) as all the variants displayed a similar melting temperature (Tm) (Supplementary Fig. S1). As the linker length did not influence the solubility or the thermal stability of the Fab_H_3 format, all further analysis was performed on the Fab_H_3 containing GS-G_4_ as the linker. We chose GS-G_4_ as the linker length as long linkers may give too much structural flexibility which can be non-optimal for downstream applications and too short linkers may result in steric or other inter-domain repulsions for Fab_H_3 molecules based on other Fabs.

To confirm that the increase in soluble yields arises from the format design i.e., removal of limitations associated with the C_H_1 domain, and not the expression conditions, we compared the CyDisCo-based soluble expression of Fab_H_3 (GS-G_4_ linker) to the Fab counterpart between an *E. coli* B and K strain (BL21 (DE3) and MG1655 respectively) in rich autoinduction media. Our results demonstrate that whereas soluble yields of the Fab_H_3 format are comparable between the two *E. coli* strains tested, they are circa two-fold higher than those of the Fab counterpart in both strains (Table 1). We also examined the CyDisCo-based soluble production of Fab_H_3 and Fab in *E. coli* BL21 (DE3) in chemically defined minimal autoinduction media. The wild type REGN10987 Fab could not be produced solubly while the Fab_H_3 format was produced in a soluble form, although with comparatively lower yields as compared to the rich media (Table 1). These findings suggest that the higher soluble yields observed for the Fab_H_3 format are a result of the format design potentially overcoming folding and production constraints of the Fab format regardless of the expression conditions.

**Table 1:** Yields of REGN10987-Fab and Fab_H_3 from 20 mL cultures in shake flasks.

Culture media	Strain	REGN10987 Fab (mg/L)	REGN10987 Fab_H_3 (mg/L)
Rich autoinduction media	BL21 (DE3)	64 ± 4	143 ± 4
	MG1655	64 ± 3	128 ± 1
Chemically defined minimal autoinduction media	BL21 (DE3)	–	46 ± 3

The protein yields are expressed in mg/L of culture media. See Supplementary Figs. S5 and S6 for uncropped SDS-PAGE gel images used for protein quantification.

### Biophysical characterization of the Fab_H_3 format

Production of disulfide-bonded therapeutic proteins in the cytoplasm of microbial hosts such as *E. coli* is often challenging due to its limited post-translational modification capabilities which often leads to protein degradation and aggregation^29^. Although employing the CyDisCo system in the *E. coli* cytoplasm allowed the production of the REGN10987 Fab and Fab_H_3 in a soluble form, with the latter in comparatively higher yields, developing an antibody format where quantity meets quality is of paramount importance. We evaluated the folding state of the antibody formats produced using a set of orthogonal analytical characterization tools prior to their functional characterization.

**Figure 3:**
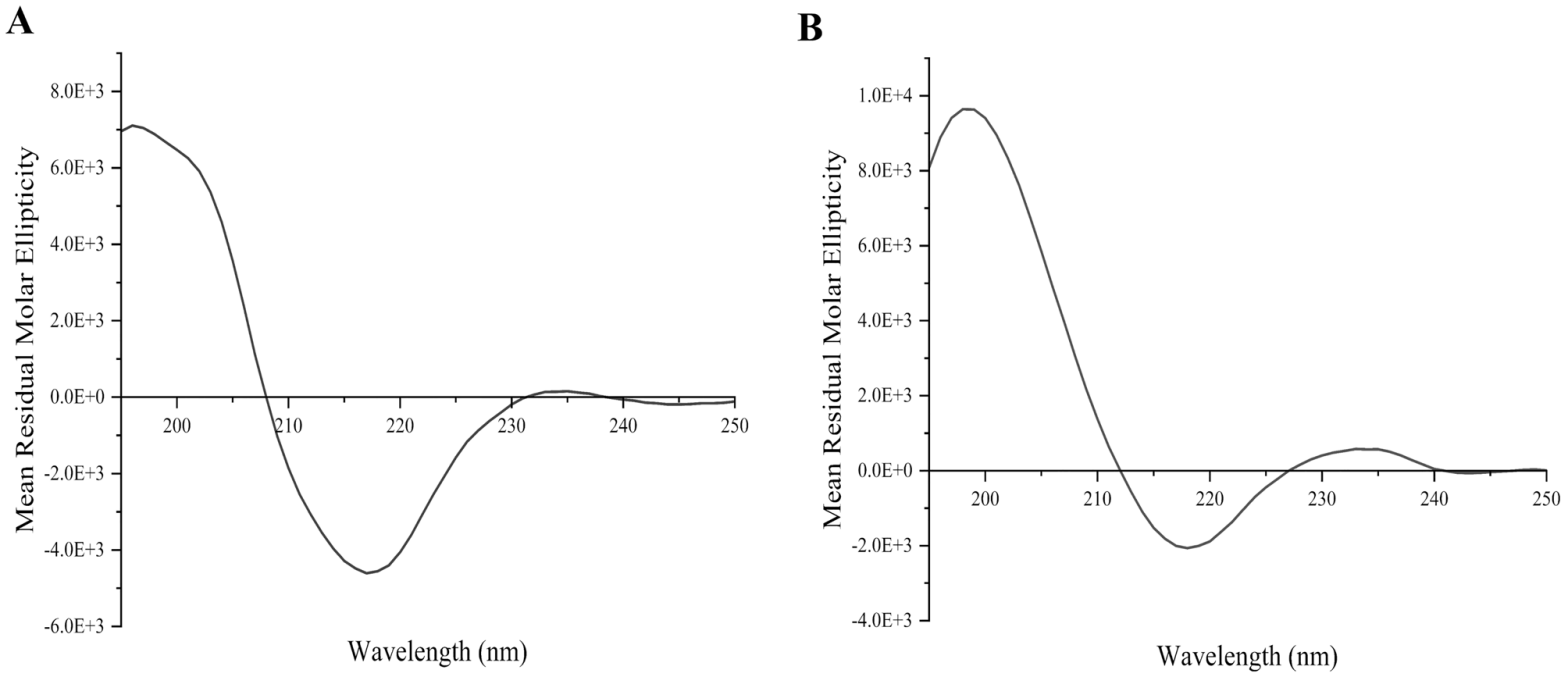
Far-ultraviolet Circular Dichroism (CD) spectra of purified proteins (**A**) REGN10987 Fab (**B**) REGN10987-based Fab_H_3. Both proteins show spectra consistent with their expected β-rich structure of immunoglobulin domains i.e., Ig-fold.

The secondary structure of the purified REGN10987 Fab and Fab_H_3 (GS-G_4_ linker) was examined using circular dichroism (CD). As both the proteins of interest are composed of domains that possess the characteristic immunoglobulin (Ig)- fold, the CD spectra of these proteins were expected to exhibit shapes typical for proteins with a high content of β structure^30^. CD analysis indicated that both the proteins exhibited far-ultraviolet (UV) CD spectra with a minima around 217 nm which is consistent with having a characteristic Ig-fold (Fig. 3A,B). The differences in the spectra can be explained as spectra of different Fab molecules have been shown to differ in terms of amplitude, shape and interception of the baseline based on aromatic side chain and/or disulfide bond chromophores^31–33^.

Further analysis by electrospray ionisation mass spectrometry (ESI–MS) of the purified REGN10987 Fab and Fab_H_3 confirmed that the proteins had the expected molecular weights (Table 2) consistent with having all cysteines in disulfide bonds. To further evaluate the presence of any free cysteines, the proteins were treated with N-ethyl maleimide (NEM) under denaturing conditions prior to mass spectrometric analysis. No increase in mass corresponding to NEM binding (+ 125 Da) was observed for the proteins of interest, thereby confirming the absence of free thiols (Table 2). A combination of CD and ESI–MS data suggests that the proteins produced using CyDisCo in the cytoplasm of *E. coli* are natively folded.

**Table 2:** Molecular weight analysis of purified REGN10987 Fab and Fab_H_3 by electrospray ionization mass spectrometry (ESI–MS) in the presence and absence of NEM under denaturing conditions.

Protein of Interest	Sample preparation	No. of cysteines	M_theor_ (Da) (oxidized)	M_exp_ (Da)	Δ mass (M_theor_ − M_exp_)
REGN10987 Fab	− NEM	10	47,892	47,894	− 2
	+ NEM	10	47,892	47,892	0
REGN10987-based Fab_H_3	− NEM	LC: 4	24,042	24,042	0
		HC: 4	26,618	26,619	− 1
	+ NEM	LC: 4	24,042	24,043	− 1
		HC: 4	26,618	26,618	0

Δ mass accounts for the mass difference between the theoretical molecular weight with disulfides formed (M_theor_) and the experimental molecular weight (M_exp_). None of the samples analyzed showed NEM binding (+ 125 Da) which suggests the absence of free thiols. The light (LC) and heavy (HC) chains of the Fab_H_3 molecule were detected as single chains instead of as a dimer since there is no covalent linkage between the chains.

The solution behaviour of purified REGN10987-Fab and Fab_H_3 was analyzed using SEC-MALS to determine the presence of native dimers as well as the absence of any degradants and high-molecular weight oligomeric species. The molecular weight determination of the two proteins under non-denaturing conditions confirmed the presence of both in a dimeric state with uniform molar mass points calculated across the elution peak to within 2% or less indicating monodisperse species (Supplementary Fig. S4A). The results obtained also demonstrate the absence of any monomeric fragments or oligomeric aggregates (Supplementary Fig. S4B).

**Figure 4:**
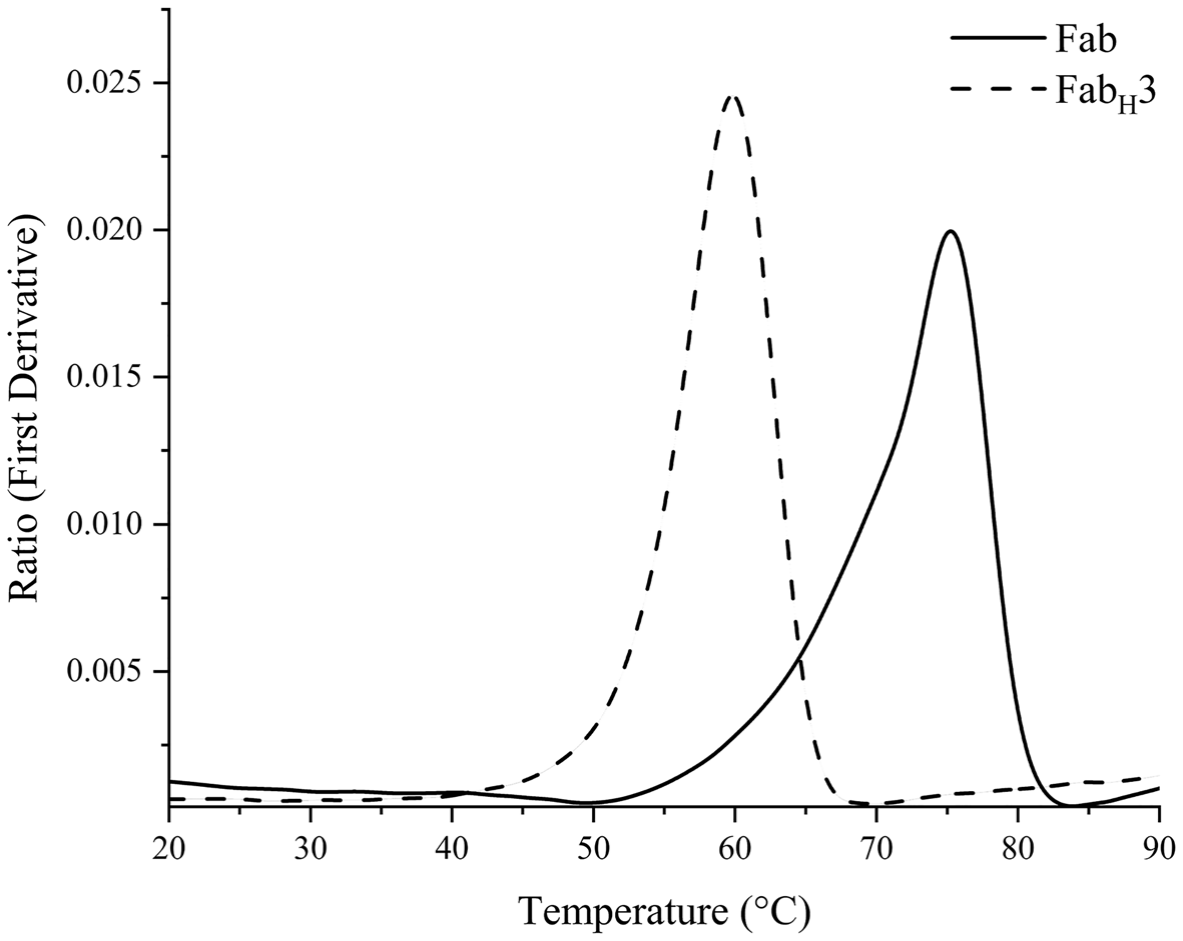
Thermal stability analysis of REGN10987 Fab and REGN10987-based Fab_H_3 using NanoDSF. The onset points for REGN10987 Fab and Fab_H_3 were found to be 50.0 °C and 42.5 °C respectively, while the final Tm values were found to be 75.1 °C and 59.9 °C respectively.

The wild type IgG_1_ C_H_3 domain is widely known to be a highly thermostable immunoglobulin domain^34,35^. However, heterodimerization is essential for a functional Fab-analogue and introducing mutations in the C_H_3 domain to allow heterodimerization in bispecific monoclonal antibodies has previously been shown to result in a reduction in the thermal stability^16,17,21,24^. We examined the thermal stability of the Fab_H_3 format produced in the cytoplasm of *E. coli* using Nano Differential Scanning Fluorimetry (NanoDSF) and it was found to be in accordance with that previously reported for the electrostatic steering based bispecific scFv-Fc fusion protein molecule produced in a mammalian expression system^16^. A co-operative unfolding was observed for the Fab_H_3 format in contrast to the non-cooperative folding observed for its Fab counterpart (Fig. 4) in accordance with previously reported unfolding characteristics of λ-Lc containing Fabs^36^. Biphasic Fab unfolding involves the loss of antigen binding activity prior to the secondary structure breakdown^37^. As observed from the thermal unfolding curves, onset of unfolding of the REGN10987 Fab occurs comparatively much earlier than the melting temperature (Fig. 4). Even though the Fab_H_3 format was found to possess a comparatively lower final Tm than the Fab, it is still within a range that allows viable therapeutic protein production as the thermal stability is in accordance with Amgen’s patented technology for generation of bispecific antibodies by electrostatic steering^16,38^.

### In vitro functional characterization of the Fab_H_3 format

One of the critical quality attributes to be investigated when developing an alternate antibody format is the interaction of the proposed molecule against the target antigen. The binding ability of an antibody is a function of the Complementarity Determining Regions (CDRs) located in the variable domains which in turn depends on the stability and association of the domain interfaces^39^.

We have previously shown that the wild type REGN10987 Fab produced using the CyDisCo system is functional and binds to the target antigen SARS-CoV-2 RBD^40^. After ensuring that the REGN10987- based Fab_H_3 produced in the cytoplasm of *E. coli* was natively folded, we empirically investigated whether the molecule developed was functionally active and compared its binding affinity with that of the REGN10987 Fab using Biolayer Interferometry (BLI). The binding affinity between the Fab or Fab_H_3 against the target antigen was assessed based on the ratio of the dissociation (k_d_) and association (k_a_) rate constants i.e., as a function of the equilibrium dissociation constant (K_D_). Five concentrations each of the Fab and Fab_H_3 were tested in parallel for their binding to SARS-CoV-2 RBD and the K_D_ values were determined using a global fit analysis based on a 1:1 interaction model.

**Figure 5:**
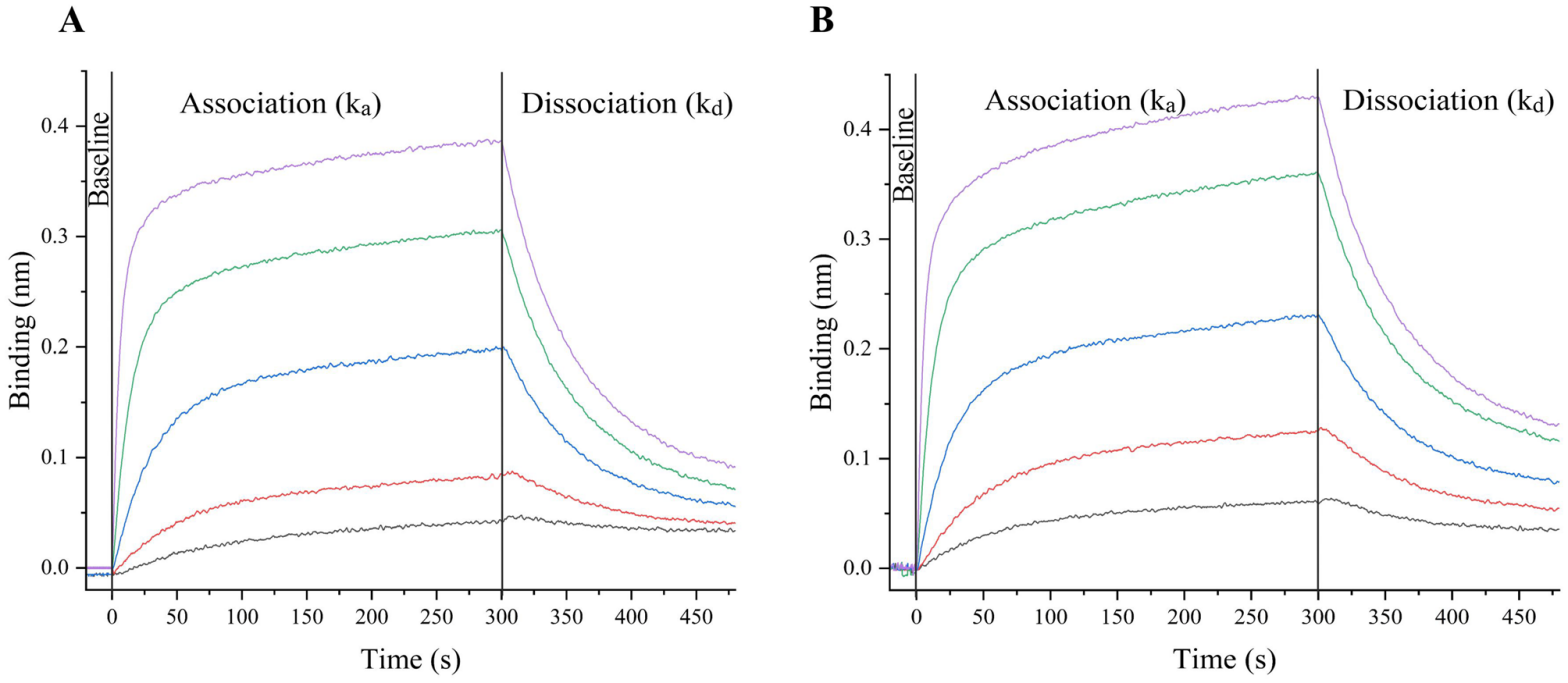
Biolayer Interferometry (BLI)-based analysis of the binding interaction between (**A**) REGN10987 Fab, (**B**) REGN10987-based Fab_H_3 and the receptor binding domain (RBD) of SARS-CoV-2. Both the antibody formats tested show concentration-dependent binding to the target antigen (Grey: 3 nM, Red: 9 nM, Blue: 27 nM, Green: 81 nM, Purple: 243 nM). The steady-state analysis curve for the antibody-antigen interaction as a function of protein concentration is shown in Supplementary Fig. S3.

Both Fab and Fab_H_3 displayed binding to the target antigen in a concentration dependent manner (Fig. 5A,B). The Fab_H_3 was found to exhibit a comparable binding affinity (K_D_ = 20 ± 2.6 nM) to its counterpart REGN10987 Fab (K_D_ = 43 ± 4.4 nM) against SARS-CoV-2 RBD. The K_D_ values observed for the REGN10987 Fab are in accordance with previous reports^40,41^ thereby validating our finding. These results suggest that the replacement of constant domains in a Fab molecule and the C_H_3 domain-based heterodimerization does not interfere with the folding and function of the variable domains such that interactions with its antigen are maintained.

## Discussion

Advances in synthetic biology leading to cellular engineering of microbial hosts have resulted in an increased acceptance of these cell factories as an alternative for producing antibody-based therapeutics^42^. While monoclonal antibodies have dominated treatment strategies against life-threatening diseases, advances in antibody engineering have led to an array of around 100 different antibody formats (see^43–45^ for comprehensive reviews on bispecific antibody formats and applications). There is no consensus on ‘one ideal format’ that is suitable for most of the desired applications, but instead the development of new formats offers a valuable source to overcome therapeutic and production bottlenecks of the current formats.

Fab antibody fragments are one of the earliest small antibody molecules studied and have demonstrated an important role in diagnostic and therapeutic interventions^46^. With the increasing demand and future potential of Fabs or their fusion proteins, there is a constant need for the ‘developability’ of this format to be produced efficiently. Building on the advances made in the development of bispecifics, the Fab_H_3 format that we introduce here has the potential to overcome certain critical limitations of Fab production in *E. coli*. The previously proven IgG_1_ C_H_3 domain heterodimerization strategy used here allows the production of heterodimeric molecules thereby preventing the light chain homodimerization limitations in wild type Fab molecules^11,47^. The limitations associated with the low intrinsic stability and degradation tendency of the C_H_1 domain in Fabs have been known to result in low-yield production of Fabs in *E. coli*^3,10,48^. These bottlenecks are potentially overcome with the strong dimerization propensity, high solubility, and stability of the IgG_1_ C_H_3 domains used in the proposed Fab_H_3 format. This supposition is validated by the higher soluble production levels of the Fab_H_3 format than the corresponding Fab counterpart. In addition, the Fab_H_3 format is biologically active and shows a comparable binding affinity to the Fab format against the target antigen. Although the wild type IgG_1_ C_H_3 domain has been reported to exhibit redox heterogeneity when produced using CyDisCo in the cytoplasm of *E. coli*^14^, the suppression of homodimerization potentially slows down the rate of folding which allows the formation of disulfide bonds and thereby native folding. This is not the first report to use IgG C_H_3 homodimers or heterodimers as scaffolds for the production of antibody formats, for e.g., scDb-C_H_3 (KiH), Di-Diabody, Minibody, etc.^49–51^. However, to the best of our knowledge, this is the first report of an alternative Fab format based on C_H_3 domain heterodimerization, one that can be produced more efficiently using the CyDisCo system in the cytoplasm of *E. coli*.

Based on the ‘proof-of-concept’ reported here, this format design can be a potential solution to Fabs which face production bottlenecks. However, the general applicability of this format has to devised empirically as it depends on the interface interactions between the variable and C_H_3 domains, and the solubility of the variable domain partners. Potential future work can involve engineering of the Fab_H_3 format to improve the thermal stability based on the previously reported ZW heterodimer variants^52^. Furthermore, protein engineering to expand the applicability of the Fab_H_3 format building on Fab-fusion proteins for a myriad of therapeutic and diagnostic applications can also be undertaken^53,54^. Mutations in the IgG_1_ C_H_3 domain have been shown to promote Fcγ receptor interaction in the absence of glycosylation^55^. Being able to be produced in a microbial host, these mutations can potentially be applied to the Fab_H_3 format to increase half-life and bring about effector functions for certain therapeutic applications^56^.

## Methods

### Cloning

All the expression vectors reported here were constructed using standard molecular biology techniques (see Supplementary Table S2 for vectors used in this study). A polycistronic gene for the REGN10987 wild type Fab was synthesized codon optimized (GenScript Biotech Corp.) for *E. coli* expression. The gene for the heavy chain with a C-terminal hexahistidine tag was placed downstream of the gene for the light chain and cloned using *Xba1-EcoR1* based restriction digestion and ligation in a modified pET23-based vector with the T7 promoter replaced by a pTac promoter to generate vector pAAT50^26,40^. Genes for the heavy and light chains of REGN10987-based Fab_H_3 variants with different linker lengths were synthesized codon optimized (GenScript Biotech Corp.) for *E. coli* expression. These genes were cloned in the same backbone vector using *Nde1-EcoR1* based restriction digestion and ligation. To generate a polycistronic vector with a similar placement of genes as the wild type Fab, the gene for the V_H_ domain fused to an IgG_1_ C_H_3 domain (K392D, K409D) with a C-terminal hexahistidine tag was placed downstream of the gene for the V_L_ domain fused to an IgG_1_ C_H_3 domain (E356K, D399K). The gene for the Fab_H_3 heavy chain was cloned into the vector containing the Fab_H_3 light chain using *Xba1/Spe1-Xho1* based restriction digestion and ligation to generate vectors pAAT202-pAAT207. Purified plasmid vectors were obtained using the E.Z.N.A Plasmid DNA Mini Kit I (Omega Bio-Tek Inc.) and purification of DNA from agarose gels was performed using the Gene/PCR DNA Fragments Extraction Kit (GeneAid Biotech), both according to manufacturers’ guidelines. All the gene inserts in the constructed vectors were fully sequenced prior to expression tests to avoid any errors in the cloned genes.

### Protein expression

Initial tests employed to screen the Fab_H_3 variants with multiple linker lengths and compare the soluble expression yields were performed in 24-deep well plates (DWPs). The polycistronic plasmid containing the genes of interest and the polycistronic plasmid containing the CyDisCo components Erv1p and PDI (pMJS205)^26^ were used to cotransform chemically competent *E. coli* BL21 (DE3) and incubated overnight at 37 °C on Lysogeny Broth (LB) agar plates containing the appropriate antibiotics (35 µg/mL chloramphenicol for pLysS derivatives and 100 µg/mL ampicillin for pET23 derivates). The transformed colonies obtained after overnight growth were used to inoculate 2 mL of LB media supplemented with 4 g/L glucose and suitable antibiotics per well in a 24-DWP covered with an oxygen permeable AirOTop (Thomson) membranes. These starter cultures were allowed to grow at 30 °C, 250 rpm (2.5 cm radius of gyration) for about 6–8 h. Expression cultures containing 3 mL autoclaved terrific broth autoinduction media (Formedium), supplemented with 0.8% glycerol and suitable antibiotics per well were seeded with the starter cultures in a 1:100 ratio. The expression cultures were grown at 30 °C, 250 rpm in 24 deep well plates covered with oxygen permeable AirOTop (Thomson) membranes to ensure efficient oxygenation for approximately 23–24 h. Soluble fraction of the cell lysate was used to perform the further purification and SDS-PAGE analysis steps to determine and compare soluble protein expression yields.

For yield quantification and comparison, pAAT50 or pAAT203 along with pMJS205 were used to cotransform chemically competent *E. coli* BL21(DE3) or MG1655 cells and grown on LB agar plates with appropriate antibiotics. Expression cultures containing 20 mL autoclaved terrific broth autoinduction media (Formedium) supplemented with 0.8% glycerol, or chemically defined minimal autoinduction media prepared in accordance with^57^ were seeded with starter cultures in a 1:100 ratio. The expression cultures were covered with oxygen permeable AirOTop (Thomson) membranes and grown in 250 mL shake flasks at 30 °C, 250 rpm for 23–24 h for rich media and ~ 40 h for chemically defined media. Final optical density values of the cultures were measured at 600 nm and were found to be in the range of 24.0–26.5 and 18.5–20.0 for *E. coli* BL21 (DE3) and MG1655 respectively in rich media, and 15.0–17.0 for *E. coli* BL21 (DE3) in chemically defined media. The protein yields were calculated from IMAC-purified fractions of the soluble lysates in triplicate via densitometry analysis using purified REGN10987-Fab or Fab_H_3 as the concentration standards. Densitometric analysis to determine target protein yields was carried out using the ImageJ software.

On completion of the expression screening at the small scale, *E. coli* BL21 (DE3) cells transformed with the vectors for REGN10987 wild type Fab (pAAT50) or Fab_H_3 with a GS-G_4_ linker (pAAT203) along with the vector for CyDisCo components (pMJS205) were grown in 1 L flasks containing 100 mL of the terrific broth autoinduction media (Formedium)These culture flasks were covered with oxygen permeable AirOTop (Thomson) membrane filters to ensure efficient oxygenation and incubated at 30 °C, 250 rpm for 23–24 h.

### Protein purification

The small-scale cultures in 24 DWPs or shake flasks were harvested using centrifugation at 6500×*g* at 4 °C and the cell pellets were resuspended in 3 mL or 20 mL of lysis buffer respectively (50 mM sodium phosphate pH 7.4, 20 μg/mL DNase, 0.1 mg/mL egg white lysozyme), incubated for 15 min at room temperature and frozen at − 20 °C. The cells were lysed by freeze thawing and as the proteins of interest contain a hexahistidine tag, they were purified with standard immobilized metal affinity chromatography (IMAC) using HisPur Cobalt Superflow Agarose (Thermo Scientific) resin under native conditions following clearance of the cell lysate by centrifugation (4000 rpm, 20 min, 4 °C). The protocol followed for Cobalt-IMAC based purification has been described in^14^.

For the large-scale cultures in 1 L flasks, the cells were harvested, and the soluble fraction of the lysate was processed for purification as described in^40^. REGN10987 wild type Fab and Fab_H_3 (GS-G_4_ linker) were purified using a combination of two chromatography steps: Nickel-based Immobilized Metal Affinity Chromatography (IMAC) and Anion Exchange Chromatography (AnEx). The detailed protocol for both these chromatography steps has been described in^40^. The purified protein obtained after the AnEx step was buffer exchanged into 20 mM phosphate, 150 mM NaCl, pH 6.5 for REGN10987 Fab and pH 6.0 for the Fab_H_3. Aliquots of the purified protein fractions were flash-freezed using liquid nitrogen and stored at − 20 °C until further analysis. The method of production and purification of mutated-truncated DsbC (mtDsbC) tagged SARS-CoV-2 Receptor Binding Domain (RBD) has been described in detail in^40^.

### Circular dichroism

Far-ultraviolet circular dichroism (CD) spectra of the purified proteins were measured using a Chirascan-Plus spectrophotometer. The scans were performed in duplicates as an average of 3 scans at 22 °C using a cuvette with a path length of 0.1 cm, spectral bandwidth and step size of 1 nm, and a scan speed of 1 nm/s. A wavelength range of 195–250 nm was used for the measurement and a high-tension (HT) voltage value of below 800 V was ensured for the scans. The final concentration of the purified protein sample (in 20 mM phosphate, 150 mM NaCl, pH 6.0/6.5) used for the analysis was 0.1 mg/mL diluted in ultrapure water. The final spectrum was obtained as an average of the scans and the blank subtracted.

### Nano differential scanning fluorimetry (NanoDSF)

Thermal stability of the proteins was assessed by NanoDSF using the Prometheus NT.48 system (NanoTemper Technologies, Germany) in triplicates. The purified proteins of interest were buffer exchanged into 20 mM sodium phosphate, pH 7.4 using a microcon-10 kDa centrifugal filter (Merck, USA) and loaded into standard capillaries (10 µL) of NanoDSF grade. These loaded samples were then subjected to programmed thermal ramping (1 °C/minute) from 20 °C to 90 °C. A dual UV detector was used to measure the fluorescence signal resulting from the thermal denaturation of the protein at 330 nm and 350 nm. The first derivative of the ratio between the two signals was used to determine the inflection point/s and the melting temperature (Tm) was calculated using the PR.ThermControl Software (NanoTemper Technologies).

### Size exclusion chromatography-multi-angle light scattering (SEC-MALS)

SEC-MALS tests were performed on a Shimadzu HPLC system with an in-line UV detector, an RI detector (Optilab, Wyatt Technology) and a MALS detector (miniDAWN, Wyatt Technology). The separation of purified proteins (1.5 mg/mL) was carried out on a Superdex™ 200 Increase 10/300 GL (Cytiva) column at a flow rate of 0.5 mL/min using 20 mM phosphate, 150 mM NaCl, pH 6.0, filtered with 0.1 μm membrane as the mobile phase. Data processing and analysis was carried out using ASTRA software v. 7.3.2.19 (Wyatt Technology).

### Mass spectrometry

Molecular weight analysis of the purified proteins was performed by Electrospray ionization mass spectrometry in combination with liquid chromatography (LC–ESI–MS) using a Q-Exactive Plus Mass Spectrometer (Waters, MA, USA). 0.5 mg/ mL of the purified protein of interest was first subjected to denaturation using 5 M guanidine hydrochloride. Post-denaturation, NEM-trapped samples were treated with 10 mM NEM, incubated at room temperature for 10 min and the alkylation reaction was then quenched with 0.5% trifluoroacetic acid (TFA) prior to analysis. For the non-NEM trapped samples, 0.5% TFA was directly added to the denatured protein samples prior to analysis. The experimental molecular weight (M_exp_) of the protein was obtained by mass spectrometry analysis and the theoretical molecular weight (M_theor_) was calculated using ExPasy ProtParam tool^58^ based on the amino acid sequence of the proteins.

### Biolayer interferometry

Prior to in vitro interaction analysis, the purified antigen mtDsbC-SARS-CoV-2 wild type was subjected to biotinylation using the protocol described in^40^. The in vitro interaction of the biotinylated antigen with wild type REGN10987 Fab and Fab_H_3 was analysed using an Octet RED384 instrument (ForteBio, USA). The assays were performed with continuous agitation at 1000 rpm, 30 °C. All the measurements were performed using 1X Phosphate Buffer Saline (PBS) Kinetics Buffer (ForteBio) in 96-well plates. After obtaining an initial baseline with the running buffer, 5 µg/mL of the biotinylated antigen was immobilized on Streptavidin (SA) Dip and Read™ Biosensors (ForteBio) for 600 s. Five different concentrations each of the purified REGN10987 Fab and Fab_H_3 were tested for their binding to the antigen. Data analysis was performed on Octet Data Analysis High Throughput (HT) software 11.0.

### Supplementary Information

Supplementary Information.

## Data Availability

The data presented in this study is contained within the article and supplementary material. The datasets are available from the corresponding author on reasonable request.
