# Supplementary material for: Design of an alternate antibody fragment format that can be produced in the cytoplasm of Escherichia coli
Source: Sci Rep. 2023 Aug 30;13:14188. doi: 10.1038/s41598-023-41525-3 (PMC10469194; doi:10.1038/s41598-023-41525-3)
Supplement: Supplementary file 1 — Supplementary Information. [file 41598_2023_41525_MOESM1_ESM.pdf]

Supporting information

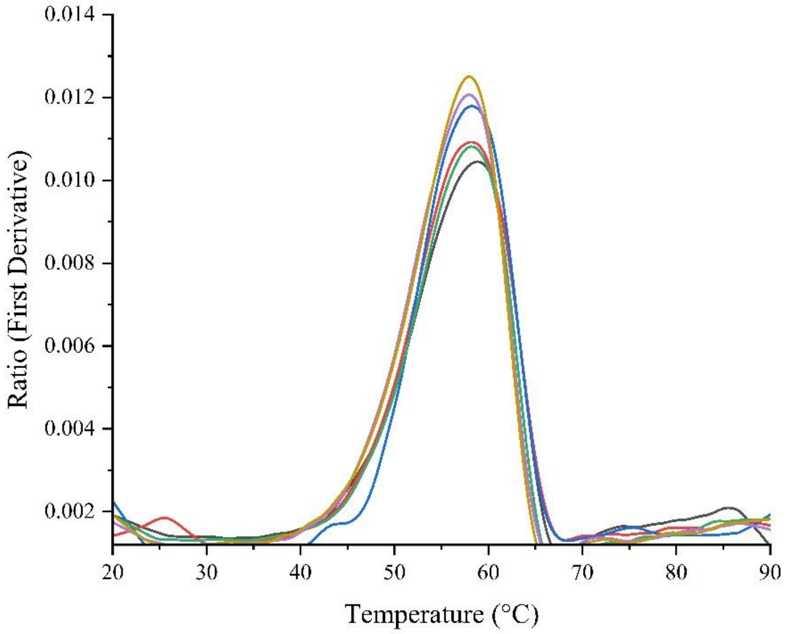

Supplementary Figure S1. Thermal stability analysis of Fab_H3 variants with varying linker lengths at the interface of the variable domains and C_H3 domains using NanoDSF. A single co-operative unfolding peak at the same melting temperature (Tm) for each of the variants suggests no influence of the linker length on the thermal stability of the Fab_H3 format. GS-G_2: Black, GS-G_4: Red, GS-G_6: Blue, GS-G_8: Green, GS-$(G_4S)_2$: Purple, GS-$(G_4S)_3$: Yellow.

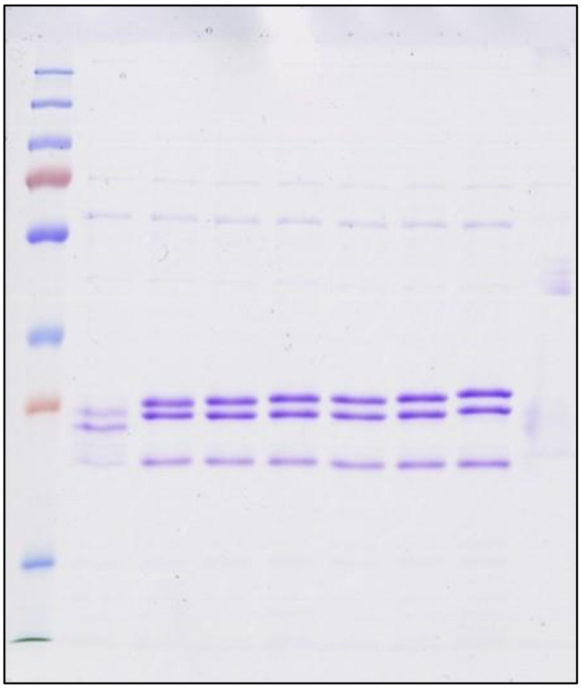

Supplementary Figure S2. Uncropped original scan of the SDS-PAGE gel image from Figure 2 in the main article.

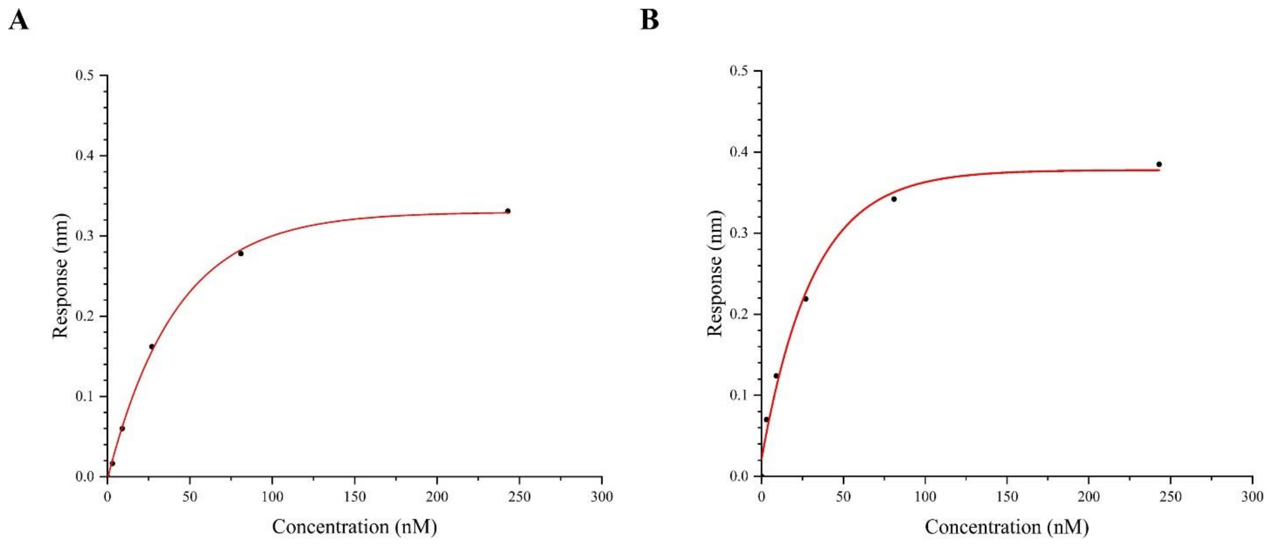

Supplementary Figure S3. Biolayer interferometry-derived steady state analysis of the binding response (nm) between **A.** REGN10987 Fab (R-square: 0.99), **B.** REGN10987-based Fab_H3 (R-square: 0.98) against SARS-CoV-2 Receptor Binding Domain (RBD) as a function of protein concentration (nM).

A **B**

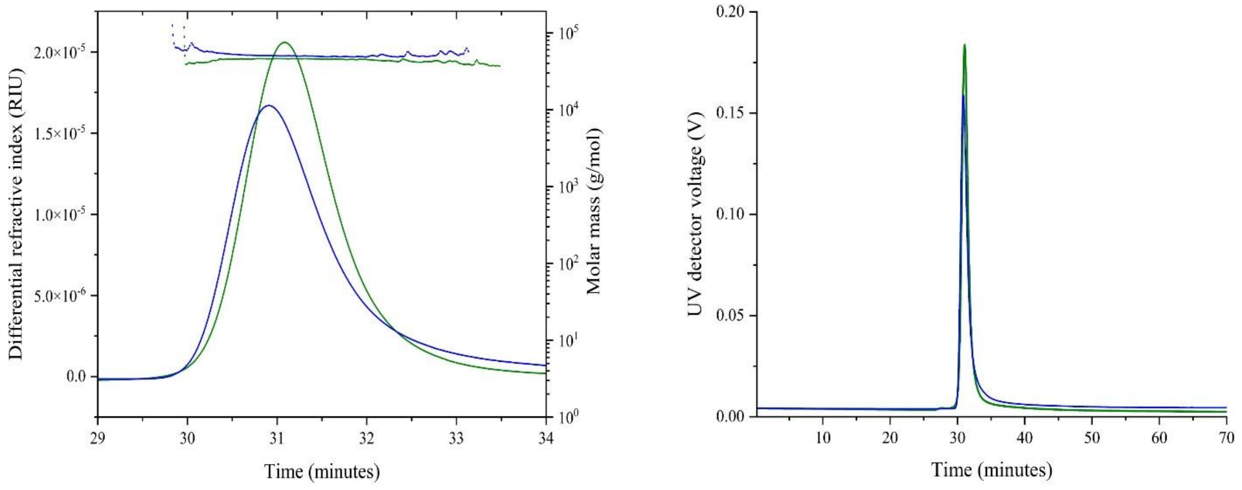

Supplementary Figure S4. SEC-MALS analysis of the purified REGN10987-Fab (Green) and Fab$_H$3 (Blue). **A.** Differential refractive index (RIU) and molar mass (g/mol) against time (minutes). Polydispersity values of 1.001 and 1.002 for REGN10987-Fab and Fab$_H$3 respectively and a uniform molar mass distribution across the peak indicate a monodisperse species. **B.** UV signal overlay of protein separation through the SEC column against time (minutes) showing a single elution peak for the dimeric proteins and the absence of any degradants or aggregates.

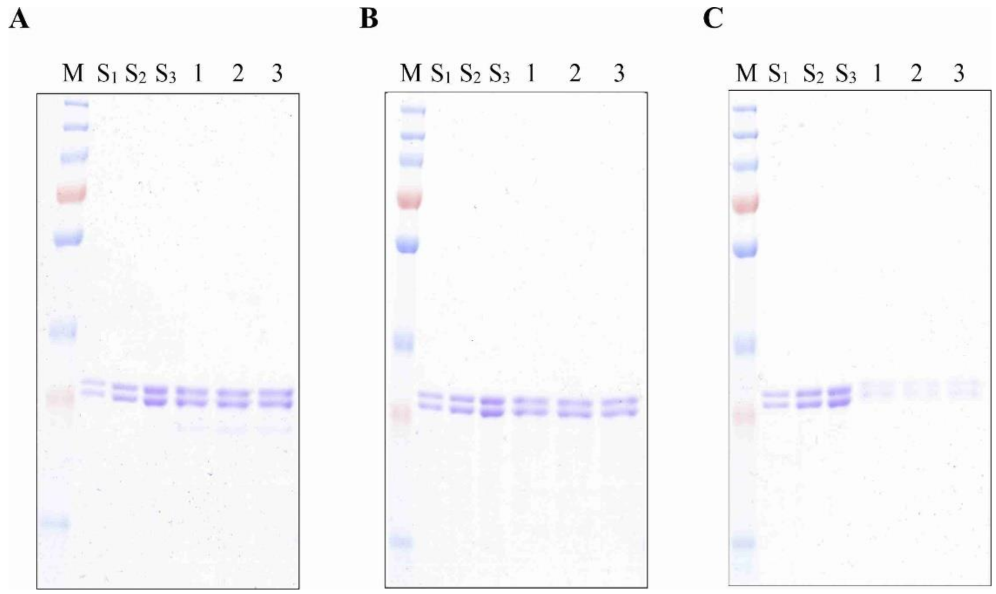

Supplementary Figure S5. Uncropped SDS-PAGE gel images used for densitometry analysis of IMAC-purified REGN10987-based Fab_H3 (GS-G_4 linker) in triplicates (1,2,3) using three different concentrations of purified Fab_H3 (S1: 0.15 mg/mL, S2: 0.225 mg/mL, S3: 0.3 mg/mL) **A.** *E. coli* BL21 (DE3) in rich media, **B.** *E. coli* MG1655 in rich media, **C.** *E. coli* BL21 (DE3) in chemically defined minimal media. (M: Protein Marker)

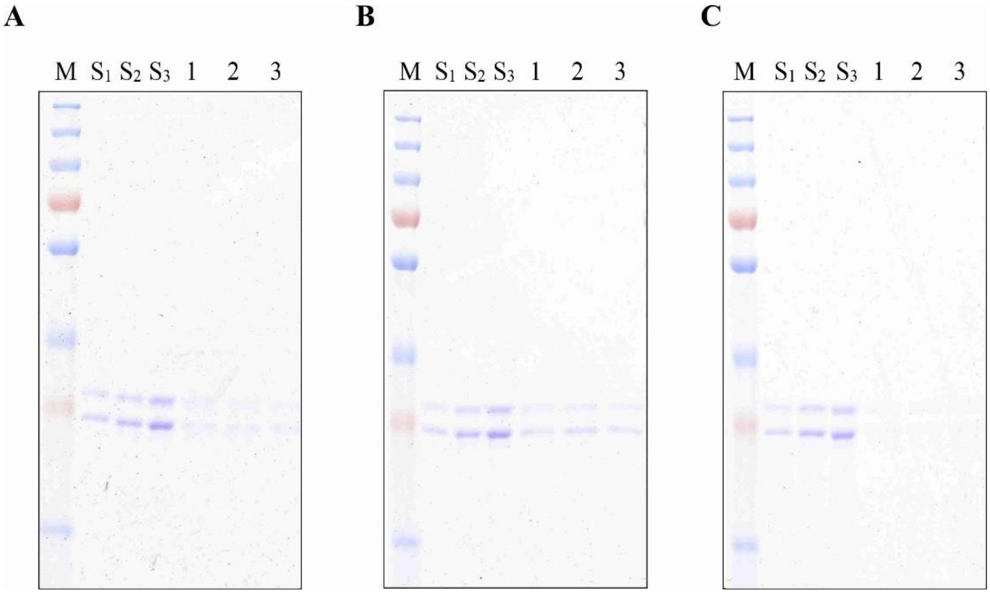

Supplementary Figure S6. Uncropped SDS-PAGE gel images used for densitometry analysis of IMAC-purified REGN10987 Fab in triplicates (1,2,3) using three different concentrations of purified REGN10987 Fab (S1: 0.15 mg/mL, S2: 0.225 mg/mL, S3: 0.3 mg/mL) **A.** *E. coli* BL21 (DE3) in rich media, **B.** *E. coli* MG1655 in rich media, **C.** *E. coli* BL21 (DE3) in chemically defined minimal media. (M: Protein Marker)

Supplementary Table S1. Amino acid sequences of the constructs used in the study

Protein name	Sequence
REGN10987<br>wild-type Fab	**Light chain:**<br>QSALTQPASVSGSPGQSITISCTGTSSDVGGYNYVSWYQQHPGKAPKLMIYDVSKRPSGV<br>SNRFSGSKSGNTASLTISGLQSEDEADYYCNSLTSISTWVFGGGTKLTVLGQPKAAPSVTL<br>FPPSSEELQANKATLVCLISDFYPGAVTVAWKADSSPVKAGVETTTPSKQSNNKYAASSY<br>LSLTPEQWKSHRSYSCQVTHEGSTVEKTVAPTECS<br>**Heavy chain:**<br>QVQLVESGGGVVQPGRSLRLSCAASGFTFSNYAMYWVRQAPGKGLEWVAVISYDGSNK<br>YYADSVKGRFTISRDNSKNTLYLQMNSLRTEDTAVYYCASGSDYGDYLLVYWGQGTLV<br>TVSSASTKGPSVFPLAPSSKSTSGGTAALGCLVKDYFPEPVTVSWNSGALTSGVHTFPAV<br>LQSSGLYSLSSVVTVPSSSLGTQTYICNVNHKPSNTKVDKKVEPKSCDGSHHHHHH
$\text{Fab}_{\text{H}}3$<br>GS-G_2 linker	**Light chain:**<br>QSALTQPASVSGSPGQSITISCTGTSSDVGGYNYVSWYQQHPGKAPKLMIYDVSKRPSGV<br>SNRFSGSKSGNTASLTISGLQSEDEADYYCNSLTSISTWVFGGGTKLTVLGGSGGQPREPQ<br>VYTLPPSRKEMTKNQVSLTCLVKGFYPSDIAVEWESNGQPENNYKTTPPVLKSDGSFFLY<br>SKLTVDKSRWQQGNVFSCSVMHEALHNHYTQKSLSLSPG<br>**Heavy chain:**<br>QVQLVESGGGVVQPGRSLRLSCAASGFTFSNYAMYWVRQAPGKGLEWVAVISYDGSNK<br>YYADSVKGRFTISRDNSKNTLYLQMNSLRTEDTAVYYCASGSDYGDYLLVYWGQGTLV<br>TVSSGSGGQPREPQVYTLPPSREEMTKNQVSLTCLVKGFYPSDIAVEWESNGQPENNYDT<br>TPPVLDSDGSFFLYSDLTVDKSRWQQGNVFSCSVMHEALHNHYTQKSLSLSPGGGSHHH<br>HHH
$\text{Fab}_{\text{H}}3$<br>GS-G_4 linker	**Light chain:**<br>QSALTQPASVSGSPGQSITISCTGTSSDVGGYNYVSWYQQHPGKAPKLMIYDVSKRPSGV<br>SNRFSGSKSGNTASLTISGLQSEDEADYYCNSLTSISTWVFGGGTKLTVLGGSGGGGQPR<br>EPQVYTLPPSRKEMTKNQVSLTCLVKGFYPSDIAVEWESNGQPENNYKTTPPVLKSDGSF<br>FLYSKLTVDKSRWQQGNVFSCSVMHEALHNHYTQKSLSLSPG<br>**Heavy chain:**<br>QVQLVESGGGVVQPGRSLRLSCAASGFTFSNYAMYWVRQAPGKGLEWVAVISYDGSNK<br>YYADSVKGRFTISRDNSKNTLYLQMNSLRTEDTAVYYCASGSDYGDYLLVYWGQGTLV<br>TVSSGSGGGGQPREPQVYTLPPSREEMTKNQVSLTCLVKGFYPSDIAVEWESNGQPENN<br>YDTTPPVLDSDGSFFLYSDLTVDKSRWQQGNVFSCSVMHEALHNHYTQKSLSLSPGGGS<br>HHHHHH

Fab$_H$3<br>GS-G_6 linker	**Light chain:**<br>QSALTQPASVSGSPGQSITISCTGTSSDVGGYNYVSWYQQHPGKAPKLMIYDVSKRPSGV<br>SNRFSGSKSGNTASLTISGLQSEDEADYYCNSLTSISTWVFGGGTKLTVLGGSGGGGGGQ<br>PREPQVYTLPPSRKEMTKNQVSLTCLVKGFYPSDIAVEWESNGQPENNYKTTPPVLKSDG<br>SFFLYSKLTVDKSRWQQGNVFSCSVMHEALHNHYTQKSLSLSPG<br>**Heavy chain:**<br>QVQLVESGGGVVQPGRSLRLSCAASGFTFSNYAMYWVRQAPGKGLEWVAVISYDGSNK<br>YYADSVKGRFTISRDNSKNTLYLQMNSLRTEDTAVYYCASGSDYGDYLLVYWGQGTLV<br>TVSSGSGGGGGGQPREPQVYTLPPSREEMTKNQVSLTCLVKGFYPSDIAVEWESNGQPE<br>NNYDTTPPVLDSDGSFFLYSDLTVDKSRWQQGNVFSCSVMHEALHNHYTQKSLSLSPGG<br>GSHHHHHH
Fab$_H$3<br>GS-G_8 linker	**Light chain:**<br>QSALTQPASVSGSPGQSITISCTGTSSDVGGYNYVSWYQQHPGKAPKLMIYDVSKRPSGV<br>SNRFSGSKSGNTASLTISGLQSEDEADYYCNSLTSISTWVFGGGTKLTVLGGSGGGGGGG<br>GQPREPQVYTLPPSRKEMTKNQVSLTCLVKGFYPSDIAVEWESNGQPENNYKTTPPVLKS<br>DGSFFLYSKLTVDKSRWQQGNVFSCSVMHEALHNHYTQKSLSLSPG<br>**Heavy chain:**<br>QVQLVESGGGVVQPGRSLRLSCAASGFTFSNYAMYWVRQAPGKGLEWVAVISYDGSNK<br>YYADSVKGRFTISRDNSKNTLYLQMNSLRTEDTAVYYCASGSDYGDYLLVYWGQGTLV<br>TVSSGSGGGGGGGGQPREPQVYTLPPSREEMTKNQVSLTCLVKGFYPSDIAVEWESNGQ<br>PENNYDTTPPVLDSDGSFFLYSDLTVDKSRWQQGNVFSCSVMHEALHNHYTQKSLSLSP<br>GGGSHHHHHH
Fab$_H$3<br>GS-$(G_4S)_2$<br>linker	**Light chain:**<br>QSALTQPASVSGSPGQSITISCTGTSSDVGGYNYVSWYQQHPGKAPKLMIYDVSKRPSGV<br>SNRFSGSKSGNTASLTISGLQSEDEADYYCNSLTSISTWVFGGGTKLTVLGGSGGGGSGG<br>GGSQPREPQVYTLPPSRKEMTKNQVSLTCLVKGFYPSDIAVEWESNGQPENNYKTTPPVL<br>KSDGSFFLYSKLTVDKSRWQQGNVFSCSVMHEALHNHYTQKSLSLSPG<br>**Heavy chain:**<br>QVQLVESGGGVVQPGRSLRLSCAASGFTFSNYAMYWVRQAPGKGLEWVAVISYDGSNK<br>YYADSVKGRFTISRDNSKNTLYLQMNSLRTEDTAVYYCASGSDYGDYLLVYWGQGTLV<br>TVSSGSGGGGSGGGGSQPREPQVYTLPPSREEMTKNQVSLTCLVKGFYPSDIAVEWESN<br>GQPENNYDTTPPVLDSDGSFFLYSDLTVDKSRWQQGNVFSCSVMHEALHNHYTQKSLSL<br>SPGGGSHHHHHH

Fab_H3<br>GS-$(G_4S)_3$<br>linker	**Light chain:**<br>QSALTQPASVSGSPGQSITISCTGTSSDVGGYNYVSWYQQHPGKAPKLMIYDVSKRPSGV<br>SNRFSGSKSGNTASLTISGLQSEDEADYYCNSLTSISTWVFGGGTKLTVLGGSGGGGSGG<br>GGSGGGGSQPREPQVYTLPPSRKEMTKNQVSLTCLVKGFYPSDIAVEWESNGQPENNYK<br>TTPPVLKSDGSFFLYSKLTVDKSRWQQGNVFSCSVMHEALHNHYTQKSLSLSPG<br>**Heavy chain:**<br>QVQLVESGGGVVQPGRSLRLSCAASGFTFSNYAMYWVRQAPGKGLEWVAVISYDGSNK<br>YYADSVKGRFTISRDNSKNTLYLQMNSLRTEDTAVYYCASGSDYGDYLLVYWGQGTLV<br>TVSSGSGGGGSGGGGSGGGGSQPREPQVYTLPPSREEMTKNQVSLTCLVKGFYPSDIAVE<br>WESNGQPENNYDTTPPVLDSDGSFFLYSDLTVDKSRWQQGNVFSCSVMHEALHNHYTQ<br>KSLSLSPGGGSHHHHHH

Supplementary Table S2. Details of plasmid vectors used in the study

Construct details		Plasmid name	Reference
**Gene 1**	**Gene 2 (Polycistronic)**		
REGN10987 Fab Light chain	Heavy chain, C-terminal His6	pAAT50	[39]
REGN10987 (V_L)- GSG_2 - IgG_1 C_H3 (E356K,D399K)	REGN10987 (V_H)- GSG_2 - IgG_1 C_H3 (K392D,K409D), C-terminal His6	pAAT202	This study
REGN10987 (V_L)- GSG_4 - IgG_1 C_H3 (E356K,D399K)	REGN10987 (V_H)- GSG_4 - IgG_1 C_H3 (K392D,K409D), C-terminal His6	pAAT203	This study
REGN10987 (V_L)- GSG_6 - IgG_1 C_H3 (E356K,D399K)	REGN10987 (V_H)- GSG_6 - IgG_1 C_H3 (K392D,K409D), C-terminal His6	pAAT204	This study
REGN10987 (V_L)- GSG_8 - IgG_1 C_H3 (E356K,D399K)	REGN10987 (V_H)- GSG_8 - IgG_1 C_H3 (K392D,K409D), C-terminal His6	pAAT205	This study
REGN10987 (V_L)- $GS(G_4S)_2$ - IgG_1 C_H3 (E356K,D399K)	REGN10987 (V_H)- $GS(G_4S)_2$ - IgG_1 C_H3 (K392D,K409D), C-terminal His6	pAAT206	This study
REGN10987 (V_L)- $GS(G_4S)_3$ - IgG_1 C_H3 (E356K,D399K)	REGN10987 (V_H)- $GS(G_4S)_3$ - IgG_1 C_H3 (K392D,K409D), C-terminal His6	pAAT207	This study
CyDisCo components		pMJS205	[26]
